# A Cautionary Case for Host Assignment Based on Broad Environmental *bla* _OXA_ Carriers

**DOI:** 10.1111/1758-2229.70327

**Published:** 2026-04-09

**Authors:** Hanseob Shin, Min Ki Jeon, Hor‐Gil Hur

**Affiliations:** ^1^ Center for Health Effects of Environmental Contamination University of Iowa Iowa City Iowa USA; ^2^ State Hygienic Laboratory University of Iowa Coralville Iowa USA; ^3^ Department of Environment and Energy Engineering Gwangju Institute of Science and Technology (GIST) Gwangju Republic of Korea; ^4^ Water Resources Research Center University of Hawaiʻi at Mānoa Honolulu Hawaii USA; ^5^ Civil, Environmental, and Construction Engineering University of Hawaiʻi at Mānoa Honolulu Hawaii USA

**Keywords:** *bla*_OXA_ gene, integrated analysis, metagenomic prediction

## Abstract

Metagenomic analyses rely heavily on contig assembly and reference databases, which can introduce substantial bias when predicting the hosts of antibiotic resistance genes (ARGs) in complex environmental microbiomes. Reference‐based metagenomic pipelines assign ARGs mostly to clinically important pathogens because publicly available genomic repositories are dominated by clinically relevant isolates. Motivated by this limitation, we investigated whether metagenomic inferences accurately reflect the true bacterial hosts of ARGs in a wastewater treatment plant, also integrating culture‐based validation. Metagenomic screening suggested that ARGs (*bla*_OXA_) were primarily associated with clinical taxa. In contrast, culture‐based screening identified a wider host distribution of *bla*_OXA_ genes. Our results imply that environmental bacteria, rather than clinically important taxa, are also hosts of *bla*_OXA_ genes. Phenotypic testing showed elevated cephalosporin minimal but no carbapenem resistance, consistent with the nature of carbapenem‐hydrolysing class D β‐lactamases. Our findings reveal that reliance on reference‐based metagenomic host prediction can underestimate the diversity of environmental ARG reservoirs. This integrated approach highlights the need for cautious interpretation of metagenomic host assignments and the importance of coupling metagenomic pipelines with culture‐dependent validation when assessing ARG ecology in the natural environments.

## Graphical abstract

Metagenomic reference‐based pipelines predominantly assigned antibiotic resistance genes to clinically associated taxa. However, cultivation‐based confirmation revealed diverse environmental hosts, demonstrating that database‐driven gene host predictions may overestimate pathogen involvement and require cautious interpretation in environmental resistance studies.

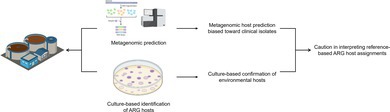

## 1 Introduction

Rapid advances in metagenomics and machine‐learning‐based host‐prediction tools have accelerated the ability to profile antibiotic resistance genes (ARGs) directly from complex environmental microbiomes (Ji et al. 2023; Derya et al. 2022; Feng et al. 2025; Su et al. 2025). Recent studies have used large‐scale reference genome collections to predict plasmid or ARG hosts from assembled contigs, enabling high‐throughput inferences that were previously impractical (Ellabaan et al. 2021; Risely et al. 2024). Also, although protein‐level classifiers such as *Kaiju* can assign a larger fraction of metagenomic reads than *k*‐mer‐based tools, they still rely heavily on reference databases, which are dominated by clinically characterised taxa (Menzel et al. 2016). Consequently, environmentally prevalent but under‐documented/uncultured bacteria are often classified as ‘unassigned’ or misattributed to clinically related species, leading to systematic bias in the representation of true microbial and ARG host diversity (Menzel et al. 2016). For example, HOTSPOT, a hierarchical transformer‐based model, predicts plasmid hosts by training on complete plasmids from RefSeq and other curated repositories to map mobile genetic elements (MGEs) across ecosystems. Similarly, language‐model approaches such as one‐class support vector machine (SVM) classifiers infer bacterial hosts using embeddings derived from known plasmid‐host pairs deposited in public databases. These tools collectively highlight how metagenomics has transformed environmental microbiology by enabling rapid, large‐scale ARG host assignment across thousands of taxa.

Despite these advances, an important limitation remains: their predictions depend heavily on reference databases that are disproportionately composed of clinical and well‐characterised taxa. Environmental microbial taxa are generally not prioritised for whole‐genome sequencing. As a result, metagenomic pipelines tend to map ARGs towards clinically important species, producing biased host assignments. Indeed, recent analyses of wastewater and soil microbiomes demonstrated that database‐driven predictions often over‐attribute β‐lactamase genes to pathogenic genera such as *Acinetobacter* or *Pseudomonas* spp. (Risely et al. 2024). This signals the need for cautious interpretation of metagenomic host predictions and highlights the importance of complementary culture‐dependent validation when assessing ARG ecology in natural environments.

Class D β‐lactamases, commonly known as OXA‐type enzymes, are serine hydrolases that hydrolyse the β‐lactam ring structure (Naas and Nordmann 1999). More than 400 OXA variants have been identified, and these enzymes exhibit widely variable hydrolysis efficiencies across β‐lactams, with a subset functioning as carbapenem‐hydrolysing class D β‐lactamases (CHDLs) that confer carbapenem resistance (Pandey et al. 2021). They have been detected in diverse environmental matrices such as wastewater pipelines, river waters and soils (Shin, Kim, Han, et al. 2023; Shin, Kim, and Hur 2023). In particular, wastewater treatment plants (WWTPs) represent a major interface between anthropogenic activities and natural environments, where *bla*_OXA_ genes can be accumulated and disseminated (Shin et al. 2021, 2022). Accordingly, qPCR and next‐generation sequencing (NGS) have been widely applied for surveillance of such ARGs in WWTPs (Raza et al. 2022). However, each method has important limitations. High‐throughput qPCR enables broad detection of ARGs, but cannot identify their bacterial hosts (Shin et al. 2022). In contrast, metagenomic sequencing can assign ARGs to putative hosts; however, this process, as described above, is biased towards reference genome databases that are disproportionately deposited for clinical relevance (Fitzpatrick and Walsh 2016). As a result, ARG distribution may be misattributed to pathogenic taxa.

To overcome these limitations, culture‐based approaches are essential to directly link ARGs with their hosts and phenotypes. In this study, we combined metagenomic surveillance with culture‐based validation to investigate *bla*_OXA_ variants and their hosts in WWTP influent and effluent. Candidate *bla*_OXA_ sequences were first identified from metagenomic datasets. Bacterial strains were then isolated, taxonomically identified and subjected to antimicrobial susceptibility tests. Targeted PCR assays confirmed the presence of bacterial *bla*_OXA_ variants identified in metagenomic analysis. This integrative strategy enabled us to reveal the bias of database‐dependent metagenomics and, at the same time, called for more cautious interpretation of metagenomic analysis, in particular, host assignment.

## 2 Materials and Methods

### 2.1 Description of Sampling Sites and Sample Collection

The Han River is one of the major rivers in South Korea and serves as the principal waterway through the Seoul metropolitan region, where more than 9 million people reside. One of the representative WWTPs, the JungRang (JR WWTP), located along the Han River, was selected for this study. This facility is one of the largest WWTPs in Seoul, South Korea, with a treatment capacity that serves more than 2.8 million residents and receives sewage from ~7000 medical facilities, including hospitals and clinics. The WWTP discharges treated effluent directly into the Han River.

The selection of JR WWTP was based on several factors. First, its coverage population is more than approximately 1/5 of the total in Seoul (~9.6 M population), which justifies the highly urbanised region. Second, the large input from healthcare facilities increases the likelihood of encountering clinically relevant ARGs, including *bla*_OXA_ variants. Third, this WWTP directly discharges effluent into the Han River, which acts as an interface between anthropogenic activities and natural environments. From this WWTP, influent (1 L) and effluent samples (4 L) were obtained in May 2018 and May 2019. Samples were transported to the laboratory within 6 h in coolers with ice. In total, four wastewater samples were analysed in this study: influent and effluent collected in May 2018 and May 2019 (*n* = 4).

### 2.2 Isolation of β‐Lactam‐Resistant Bacteria

Upon arrival at the laboratory, 1 L of wastewater samples was pre‐filtered through 10 μm pore‐size membranes, followed by 0.22 μm pore‐size membranes (Advantec, Japan). Each membrane was placed into 10 mL of Mueller–Hinton (MH) broth and thoroughly vortexed to resuspend bacteria. The suspension was serially diluted up to 10^−4^ fold. Aliquots (100 μL) of each dilution were spread onto MH agar plates supplemented with either cephalexin (32 μg/mL) or amoxicillin (32 μg/mL). The antibiotic concentrations were determined as twice the Clinical and Laboratory Standards Institute (CLSI) clinical breakpoints for *Enterobacterales*, as WWTP samples harbour diverse bacterial organisms, including *Enterobacterales* as well as *Pseudomonas* spp., *Acinetobacter* spp., *Aeromonas* spp. and other genera. To obtain resistant isolates potentially carrying *bla*_OXA_ variants rather than to determine clinical MICs, higher concentrations were applied to ensure the recovery of antibiotic‐resistant bacteria (ARB). Plates were incubated at 28°C for 48 h. Colonies that grew on agar plates were randomly chosen and sub‐cultured on fresh MH agar containing the corresponding antibiotics, followed by incubation at 28°C for 24 h to obtain single colonies. Isolates were preserved in 80% glycerol stocks at −80°C for downstream analyses.

### 2.3 DNA Extraction

Influent (1 L) and effluent (1 L) samples were pre‐filtered through 10 μm Nylon Net membranes (Merk, Germany) and then filtered through 0.22 μm mixed cellulose ester membranes (Advantec, Japan) to accumulate bacterial cells. The filter membranes were immediately stored at −80°C for further work. Genomic DNA (gDNA) was extracted from the filters using the PowerWater Kit (QIAGEN, Hilden, Germany). The concentration of genomic DNA was determined by using Nanodrop (MicroDigital, South Korea).

### 2.4 Shotgun Sequencing and Metagenomic Analysis

The extracted DNA was sent to Macrogen Inc. (Seoul, South Korea) for high‐throughput sequencing using an Illumina HiSeq 4000 platform (2 × 150 bp). On average, 20 Gb of metagenomic reads were generated for each sample. Quality control of all the metagenomic data sets was performed prior to the downstream analysis. FastQ Quality Control Software (FaQCs v2.10) (Lo and Chain 2014) was used for quality control, by removing the reads that contained more than three ambiguous nucleotides, low‐quality reads with lengths of less than 100 bp, adapter sequences and the reads with quality scores below 30. For downstream analysis, all the clean metagenomic reads were assembled into contigs with MEGAHIT (v1.2.9) using default parameters (Li et al. 2015). All contigs with lengths less than 1000 bp were removed for downstream analysis. We applied conservative filtering to minimise potential assembly artefacts because assembled contigs were used for downstream ARG‐host linkage analyses. All downstream analyses were restricted to contigs > 1000 bp and high‐confidence ARG annotations. Basic assembly statistics (e.g., total assembly size, N50, mean contig length) were not retained from the original assembly outputs and therefore could not be reported; accordingly, interpretations based on contig‐level inference were framed conservatively. Contig coverage was calculated as Hits Per Million reads (HPM) by an in‐house script of edge using samtools (v1.15), bwa (v0.7.17) and bowtie2 (v2.4.5) algorithm (Li et al. 2017). Open reading frames (ORF) were predicted in contigs using Prodigal (v2.6.3) with the ‐c option as closed end by not allowing ORF to run off the edges (Hyatt et al. 2010). ORFs were annotated against the ARGs database (CARD) (https://card.mcmaster.ca/) using BLASTP (v2.12.0+), with filtering criteria of identity of 80%, e‐value of 1e−10 and alignment length of 35 aa, and were classified based on class of antibiotics (Kanger et al. 2019). Gephi (v0.9.7) was used to construct a co‐occurrence network graph of ARGs and host bacteria (Bastian et al. 2009).

### 2.5 Selection of Candidate *bla*_OXA_ Genes and Primer Design

A total of 20 variants of seven *bla*_OXA_ groups were identified from metagenomic analysis as follows: *bla*_OXA‐10_ (*bla*_OXA‐1_ and *bla*_OXA‐4_), *bla*_OXA‐2_, *bla*_OXA‐5_ (*bla*_OXA‐129_), *bla*_OXA‐10_ (*bla*_OXA‐17_, *bla*_OXA‐334_, *bla*_OXA‐10_, *bla*_OXA‐209_, *bla*_OXA‐101_ and *bla*_OXA‐13_), *bla*_OXA‐211_ (*bla*_OXA‐211_ and *bla*_OXA‐309_), *bla*_OXA‐228_ (*bla*_OXA‐274_), *bla*_OXA‐296_ (*bla*_OXA‐296_) and unclassified group (*bla*_OXA‐333_, *bla*_OXA‐119_, *bla*_OXA‐205_, *bla*_OXA‐322_, *bla*_OXA‐9_ and *bla*_OXA‐347_). They were selected for primer design and genotypic resistance testing. Reference sequences for each *bla*_OXA_ variant were retrieved from the NCBI nucleotide database. Primers were designed using NCBI Primer‐BLAST with the following parameters: target amplicon size of 200–700 bp, primer melting temperature (*T*_m_) between 55°C and 62°C, GC content of 40%–60%, and minimal predicted secondary structures (e.g., hairpin loop). Primer specificity was evaluated in silico against the NCBI database to minimise off‐target amplification. Reference sequence accession numbers and annealing temperatures used in PCR were based on predicted primer *T*_m_ values and are listed in Table S1.

### 2.6 Phenotypic and Genotypic Susceptibility Testing

#### 2.6.1 Phenotypic Testing

E‐test (E‐strip, bioMérieux, France) was performed to evaluate antibiotic susceptibility. Briefly, bacterial suspensions adjusted to the 0.5 McFarland standard were inoculated onto MH agar plates, and E‐test strips containing cefotaxime (CTX), ceftazidime (CAZ), cefoxitin (FOX), meropenem (MRP) and imipenem (IMI) were applied to the surface. Plates were incubated at 37°C for 24 h, and minimum inhibitory concentrations (MICs, μg/mL) were read at the point where the elliptical inhibition zone crossed the strip, following the manufacturer's instructions. Results were interpreted according to CLSI M100 guidelines (Wayne 2010). Interpretive criteria were applied as a standardised reference framework for comparative purposes rather than definitive clinical categorisation because CLSI M100 breakpoints are primarily defined for clinically relevant organisms.

#### 2.6.2 Genotypic Testing

Bacterial isolates were also screened for the detection of *bla*_OXA_ genes. Bacterial DNA templates were prepared by boiling cell suspensions in 0.05 N NaOH at 95°C for 10 min, followed by a 1/10 dilution with nuclease‐free water. PCR assays targeting *bla*_OXA_ genes were performed using primer sets and annealing temperatures listed in Table S1. Primers were designed using the NCBI Primer‐BLAST tool, with the NCBI reference sequence of target *bla*_OXA_ genes as the template. Each reaction was carried out in a total volume of 20 μL using AccuPower PCR PreMix (Bioneer, Korea), which contained Taq DNA polymerase, dNTPs, reaction buffer and tracking dye. Template DNA (2 μL) and 0.5 μM of each primer were added to the premix according to the manufacturer's instructions, and the remaining volume was adjusted with sterile distilled water to a final reaction volume of 20 μL. Thermal cycling was performed with an initial denaturation at 95°C for 5 min, followed by 30 cycles of denaturation at 95°C for 30 s, annealing at the primer‐specific temperature (Table S1) for 30 s, extension at 72°C for 45 s, and a final extension at 72°C for 7 min. The amplicons were observed by gel electrophoresis (1.5% agarose gel, 80 V for 20 min) and confirmed by Sanger sequencing at Macrogen (Daejeon, South Korea).

## 3 Results

### 3.1 *Acinetobacter* spp. as the Dominant Reservoir of *bla*_OXA_ Genes by Metagenomic Analysis

**FIGURE 1:**
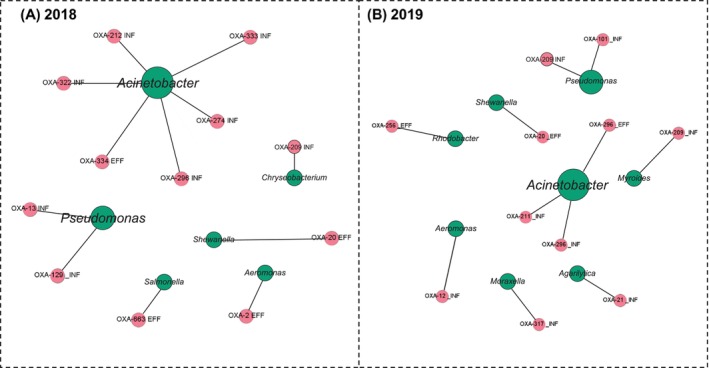
Network analysis of predicted bacterial hosts carrying *bla*_OXA_ variants in JR wastewater samples (2018–2019). Host–gene association networks constructed from metagenomic predictions in (A) 2018 and (B) 2019. Green nodes indicate predicted bacterial genera, and pink nodes denote distinct *bla*_OXA_ variants identified from metagenomic assemblies. Node size is proportional to the number of connections. Edges connect *bla*_OXA_ variants to their predicted bacterial hosts, with node labels indicating the specific variant and sample type. EFF: effluent; INF: influent.

To characterise the predicted host distribution of *bla*_OXA_ genes, metagenomic assemblies from four wastewater samples (2018 INF, 2018 EFF, 2019 INF and 2019 EFF) were analysed separately. Across these samples, a total of 20 *bla*_OXA_ variants representing seven enzyme complex groups were detected (Table 1). Among genus‐level assignments (Figure 1), *Acinetobacter* sp. was predicted as the host for seven variants, accounting for the largest proportion of identified host assignments. *Pseudomonas* sp. was associated with three variants, whereas the other genera, including *Rhodobacter*, *Aeromonas* and *Chryseobacterium*, collectively accounted for four variant–host associations. In addition to these dominant hosts, several environmental genera were linked to distinct *bla*_OXA_ genes, including *bla*_OXA‐209_, *Chryseobacterium* (*bla*_OXA‐209_), *Rhodobacter* (*bla*_OXA‐256_), *Aeromonas* (*bla*_OXA‐119_) and *Riemerella* (*bla*_OXA‐347_) (Figure 1).

**TABLE 1:** Metagenomic analysis of the diverse distribution of blaOXA genes and their predicted host bacteria in influent and effluent samples collected from the JR wastewater treatment plant (WWTP) in 2018–2019.

Enzyme complex group	Enzyme	Host genus	Water source	Year
*bla*_OXA‐1_	*bla*_OXA‐1_	N	INF	2019
	*bla*_OXA‐4_	N	EFF	2018
*bla*_OXA‐2_	*bla*_OXA‐2_	N	EFF	2018
		N	INF	2018
		N	EFF	2019
*bla*_OXA‐5_	*bla*_OXA‐129_	*Pseudomonas*	INF	2018
		N	INF	2018
		*Pseudomonas*	INF	2019
		N	INF	2019
*bla*_OXA‐10_	*bla*_OXA‐17_	N	INF	2019
		N	EFF	2019
	*bla*_OXA‐334_	*Acinetobacter*	INF	2019
		*Acinetobacter*	EFF	2018
	*bla*_OXA‐256_	*Rhodobacter*	EFF	2019
	*bla*_OXA‐10_	N	INF	2018
	*bla*_OXA‐209_	*Acinetobacter*	INF	2018
		*Chryseobacterium*	INF	2019
	*bla*_OXA‐101_	*Pseudomonas*	INF	2019
	*bla*_OXA‐13_	*Pseudomonas*	INF	2018
*bla*_OXA‐211_	*bla*_OXA‐211_	*Acinetobacter*	INF	2018
	*bla*_OXA‐309_	*Acinetobacter*	INF	2018
*bla*_OXA‐228_	*bla*_OXA‐274_	*Acinetobacter*	INF	2018
		*Acinetobacter*	INF	2018
		*Acinetobacter*	INF	2018
*bla*_OXA‐296_	*bla*_OXA‐296_	*Acinetobacter*	INF	2018
		*Acinetobacter*	EFF	2018
		*Acinetobacter*	INF	2019
		*Acinetobacter*	EFF	2019
Unclassified	*bla*_OXA‐333_	*Acinetobacter*	EFF	2018
	*bla*_OXA‐119_	*Aeromonas*	INF	2019
	*bla*_OXA‐205_	N	INF	2019
	*bla*_OXA‐322_	*Acinetobacter*	INF	2019
	*bla*_OXA‐9_	N	EFF	2018
		N	EFF	2019
	*bla*_OXA‐347_	N	INF	2019

*Note:* Water source: influent (INF) or effluent (EFF). In host genus, N represents ‘not identified’.

### 3.2 Culture‐Based Recovery of *bla*_OXA_ ‐Positive Isolates

To validate metagenomic host predictions, 192 antibiotic‐resistant isolates (48 per sample) were screened by PCR for *bla*_OXA_ genes. Nineteen isolates (19/192; 9.9%) were confirmed to carry *bla*_OXA_ genes. These isolates belonged to eight genera and spanned four *bla*_OXA_ families (*bla*_OXA‐2_, *bla*_OXA‐10_, *bla*_OXA‐129_ and *bla*_OXA‐296_) (Table 2). Across the four wastewater samples (2018 influent, 2018 effluent, 2019 influent and 2019 effluent), *bla*_OXA_‐positive isolates were recovered from both influent and effluent in both years. In the 2018 influent, positive isolates included *Aeromonas* spp. (*n* = 2; *bla*_OXA‐296_), *Myroides* spp. (*n* = 3; *bla*_OXA‐2_), *Rhodobacter* sp. (*n* = 1; *bla*_OXA‐2_) and *Chryseobacterium* sp. (*n* = 1; *bla*_OXA‐129_). Effluent samples also contained *bla*_OXA_‐positive *Moraxella* sp. in 2018 (*n* = 1; *bla*_OXA‐129_), and, in 2019, *Acinetobacter* spp. (*n* = 2; *bla*_OXA‐2_) and *Enhydrobacter* spp. (*n* = 4; two *bla*_OXA‐10_ and the other two *bla*_OXA‐129_). The other five *bla*_OXA_‐positive isolates were not identified by 16S rDNA amplicon sequencing. Those carried *bla*_OXA‐2_, *bla*_OXA‐10_ or *bla*_OXA‐129_ (Table 2). Considering variant distribution in environmental host bacteria, four *bla*_OXA_ families were observed: *bla*_OXA‐2_ (*Myroides* spp., *Rhodobacter* sp., *Acinetobacter* spp. and ND), *bla*_OXA‐10_ (*Enhydrobacter* spp. and ND), *bla*_OXA‐129_ (*Chryseobacterium* sp., *Moraxella* sp., *Enhydrobacter* spp. and ND) and *bla*_OXA‐296_ (*Aeromonas* spp.) (Table 2).

**TABLE 2:** Bacterial isolates carrying *bla*_OXA_ genes recovered from influent and effluent samples of the JR wastewater treatment plant (2018–2019).

Host genus	ID	OXA enzymes	Source (year)	Total
*Aeromonas*	ID01	OXA‐296	INF (2018)	2
*Myroides*	ID02	OXA‐2	INF (2018)	3
*Rhodobacter*	ID03	OXA‐2	INF (2018)	1
*Acinetobacter*	ID04	OXA‐2	EFF (2019)	2
*Chryseobacterium*	ID05	OXA‐129	INF (2018)	1
*Moraxella*	ID06	OXA‐129	EFF (2018)	1
*Enhydrobacter*	ID07	OXA‐10	EFF (2019)	2
*Enhydrobacter*	ID08	OXA‐129	EFF (2019)	2
Unidentified	ND	OXA‐2, OXA‐129, OXA‐10	INF (2019), EFF (2018, 2019)	5
Total				19

*Note:* Bacterial isolates were cultured and identified from wastewater influent (INF) and effluent (EFF) samples collected in 2018 and 2019. Each isolate was assigned a unique ID (ID01–ID08). Isolates of the same genus, recovered from the same sampling location, were considered clonal and therefore assigned a single ID. The *bla*_OXA_ genes were confirmed by PCR and sequencing, and host genera were determined by 16S rRNA gene sequencing.

Abbreviation: ND, not defined.

### 3.3 Phenotypic Resistance Profiles of *bla*_OXA_ ‐Positive Isolates

*E. coli* ATCC25922 was used for quality control; however, reference strains carrying *bla*_OXA‐2_, *bla*_OXA‐10_, *bla*_OXA‐129_ and *bla*_OXA‐296_ are currently not available from public repositories, limiting the use of standardised positive controls. The control strain *E. coli* ATCC25922 displayed low MICs across all five antimicrobials, serving as a susceptible reference (cefotaxime [CTX] 0.03–0.12 μg/mL; ceftazidime [CAZ] 0.125–0.5 μg/mL; cefoxitin [FOX] 1–2 μg/mL; meropenem [MRP] 0.016–0.125 μg/mL; imipenem [IMI] 0.125–0.25 μg/mL).

Environmental isolates showed similar resistance trends to five antibiotics (Table 3). *Acinetobacter* spp. (ID01 and ID02), *Myroides* spp. (ID11–13) and *Rhodobacter* sp. (ID14), all carrying *bla*_OXA‐2_, showed low MICs of CTX and CAZ (0.5–2 μg/mL and 2–8 μg/mL, respectively), but relatively high FOX resistance (> 256 μg/mL in *Myroides* spp. and *Acinetobacter* spp., 64 μg/mL in *Rhodobacter* sp.). *Aeromonas* spp. (ID03 and ID04) carrying OXA‐296 displayed cephalosporin resistance (CTX 1 μg/mL; CAZ 2 μg/mL; FOX > 256 μg/mL) while susceptible to carbapenems (IMI and MRP). Strains carrying *bla*_OXA‐129_, such as *Chryseobacterium* sp. (ID05) and *Moraxella* sp. (ID10), also exhibited low CTX/CAZ MICs (1–2 and 2–4 μg/mL) but resistance to FOX (> 256 μg/mL). Two *Enhydrobacter* spp. isolates, harbouring *bla*_OXA‐10_ (ID07) and *bla*_OXA‐129_ (ID08), displayed different patterns, while both showed susceptibility to carbapenems (IMI and MRP). The *bla*_OXA‐10_ positive strain (ID07) showed CTX, CAZ and FOX resistance (> 32 μg/mL, 8 μg/mL and > 256 μg/mL, respectively). In contrast, the *bla*_OXA‐129_‐positive strain (ID08) exhibited relatively lower resistance to CTX (4 μg/mL), CAZ (8 μg/mL) and FOX (32 μg/mL) compared to ID07. Phenotypic resistance testing was not performed for unidentified isolates.

**TABLE 3:** Minimal inhibitory concentrations (MICs, μg/mL) of *bla*_OXA_‐carrying isolates against β‐lactam antibiotics. MICs were determined using the E‐test according to CLSI M100 interpretive standards.

OXA type	Strains	MICs				
		CTX	CAZ	FOX	MRP	IMI
Control	*E. coli strain* ATCC25922	0.03	0.125	2	0.016	0.125
OXA‐296	*Aeromonas* spp. (ID01)	1	2	> 256	0.25	0.5
OXA‐2	*Myroides* spp. (ID02)	2	8	> 256	0.25	0.5
OXA‐2	*Rhodobacter* (ID03)	0.5	4	64	0.125	0.25
OXA‐2	*Acinetobacter* (ID04)	1	2	> 256	0.25	0.5
OXA‐129	*Chryseobacterium* (ID05)	2	4	> 256	0.125	0.25
OXA‐129	*Moraxella* (ID06)	1	2	> 256	0.125	0.5
OXA‐10	*Enhydrobacter* (ID07)	> 32	8	> 256	0.016	0.38
OXA‐129	*Enhydrobacter* (ID08)	4	8	32	0.016	0.38

Abbreviations: CAZ, ceftazidime; CTX, cefotaxime; FOX, cefoxitin; IMI, imipenem; MRP, meropenem.

## 4 Discussion

Publicly available genomic repositories such as NCBI are heavily shaped by clinical priorities because researchers tend to focus on clinically relevant pathogens for reasons of cost, labour efficiency and translational impact. Consequently, whole‐genome sequencing efforts are disproportionately focused on clinically relevant organisms. As a result, metagenomic mapping against reference databases may generate host predictions inherently biased towards clinical organisms, particularly clinically significant pathogens. This bias motivated the present study, in which we compared database‐driven predictions with direct cultivation of viable environmental bacteria and assessed the phenotypic resistance associated with the presence of the *bla*_OXA_ genes.

Our culture‐dependent and culture‐independent analyses revealed distinct distribution patterns of *bla*_OXA_ genes and their associated hosts. Metagenomic screening detected only non‐CHDL *bla*_OXA_ variants in both influent and effluent compartments, with no evidence of CHDLs. These metagenomic predictions may reflect reference database composition towards clinically important pathogens. In contrast, our cultivation‐based workflow, although limited in the number of isolates obtained, recovered *bla*_OXA_‐positive bacteria that were largely non‐pathogenic environmental species rather than clinically associated hosts. Nonetheless, the cultured isolates carried the same non‐CHDL variants predicted by metagenomic analysis, including *bla*_OXA‐2_, *bla*_OXA‐10_, *bla*_OXA‐129_ and *bla*_OXA‐296_.

Among the *bla*_OXA_ variants identified, *bla*_OXA‐2_ was the most frequently detected, appearing in *Acinetobacter*, *Myroides*, *Rhodobacter* and several unidentified isolates while the host of *bla*_OXA‐2_ was predicted as *Aeromonas*. This broad distribution of *bla*_OXA‐2_ across phylogenetically diverse genera suggests its mobility through horizontal gene transfer, previously reported to be associated with plasmids such as IncF‐type replicons in clinical and environmental isolates (Hamidian et al. 2014). In contrast, *bla*_OXA‐10_, traditionally associated with plasmid‐mediated dissemination in Gram‐negative pathogens (Maurya et al. 2017), was found in *Enhydrobacter* and unidentified isolates, indicating that this variant may also circulate in non‐clinical, environmental bacteria. The *bla*_OXA‐129_ was detected in *Chryseobacterium*, *Enhydrobacter*, *Moraxella* and an unidentified bacterium, suggesting its ecological versatility and potential adaptation to multiple environmental bacteria. The *bla*_OXA‐296_ was detected in association with *Acinetobacter* across influent and effluent samples in both years, whereas culture‐based isolation recovered *bla*_OXA‐296_ only from *Aeromonas* spp. Because of the limited number of cultured isolates and constraints in gene‐host linkage from short‐read metagenomic assemblies, these findings should be interpreted cautiously rather than as definitive evidence of a broader host range. Although our culture‐dependent approach has limited numbers of isolates and target *bla*_OXA_ variants, these findings may expand the known host range of *bla*_OXA_ genes and, by integrating culture‐dependent validation with metagenomic prediction, help overcome inherent biases in database‐dependent metagenomic host inference.

From a phenotypic perspective, most isolates displayed modestly elevated MICs against cephalosporins, compared to the susceptible quality control strain ( *E. coli* ATCC 25922) but did not exhibit carbapenem resistance. FOX resistance was consistently high across nearly all isolates, regardless of host genus or *bla*_OXA_ variant, whereas CTX and CAZ resistance levels varied depending on the gene‐host combination. Importantly, carbapenem MICs (IMI and MRP) remained low, suggesting that those genes did not contribute to carbapenem resistance as non‐CHDLs. The lack of whole‐genome sequencing in this study represents a limitation, as co‐resident resistance determinants or genetic context could not be fully resolved.

Nevertheless, the comparison between metagenomic prediction and culture‐based validation gave us a key implication. Whereas the public database‐driven approach emphasised clinically relevant taxa such as *Acinetobacter* and *Pseudomonas*, our cultivation efforts revealed a broader set of environmental hosts harbouring *bla*_OXA_ genes. This demonstrates that the environmental reservoir of ARGs is not restricted to clinically significant taxa but also includes diverse non‐clinical bacterial taxa. The implication is that metagenome‐based resistance surveillance pipelines relying solely on database‐based host prediction may underestimate the potential of environmental microbial communities as reservoirs in natural systems. By integrating both metagenomic inference and cultivation‐based verification, we show that the host range of *bla*_OXA_ genes is wider than previously recognised and that environmental bacteria have the potential to contribute to the dissemination potential of β‐lactam resistance genes.

## 5 Limitation of This Study

We acknowledge that our study did not include targeted isolation of the genera predicted by metagenomic analysis (e.g., *Acinetobacter* and *Pseudomonas*). Wastewater samples were fully processed during initial culturing and DNA extraction, precluding additional selective recovery. Moreover, because our metagenomic data were generated using short‐read shotgun sequencing, strain‐resolved assembly and definitive gene–host linkage were limited. Thus, host assignment relied on contig‐level classification against reference databases, which may favour well‐represented clinical taxa. Our findings should therefore be interpreted as highlighting potential bias in database‐dependent host inference rather than demonstrating definitive potential misattribution.

Whole‐genome sequencing of the cultured isolates would allow direct comparison of *bla*_OXA_ genomic context between metagenomic assemblies and cultured strains, including plasmid replicon typing and structural analysis. Because such sequencing was not performed in this study, horizontal gene transfer mechanisms and plasmid associations remain speculative. Future integration of isolate whole‐genome sequencing and long‐read metagenomics will be necessary to resolve gene context and confirm host‐gene linkage. In addition, quantitative abundance estimates were not calculated, and ecological interpretation was therefore limited to comparative presence and host distribution patterns because *bla*_OXA_ detection was based on assembled contigs rather than direct read mapping.

We acknowledge that interspecies variability in intrinsic β‐lactam susceptibility may influence MIC values independent of *bla*_OXA_ carriage. Because species‐matched *bla*_OXA_‐negative controls or isogenic mutants were not included, our phenotypic results should be interpreted as descriptive rather than demonstrating direct causality between gene presence and resistance phenotype. In addition, conclusions regarding host misassignment should be interpreted cautiously because direct allele‐level comparison between metagenomic contigs and cultured *bla*_OXA_ sequences was not performed.

## 6 Conclusion

This study was motivated by the hypothesis that publicly available genomic databases may be biased towards clinically relevant isolates, leading to an incomplete representation of *bla*_OXA_ diversity in natural environments. By combining metagenomic prediction with culture‐based validation, we revealed that non‐carbapenemase *bla*_OXA_ variants, particularly *bla*_OXA‐2_, *bla*_OXA‐10_, *bla*_OXA‐129_ and *bla*_OXA‐296_, were dominant and widely distributed across diverse bacterial hosts in the JR WWTP along the Han River, South Korea. These findings could highlight the potential influence of clinical overrepresentation based on reference datasets. Although not all OXA‐producing bacteria identified in this study are opportunistic pathogens, horizontal transfer and mutations (evolution into CHDLs) of *bla*_OXA_ genes could promote the emergence of highly resistant strains. Therefore, metagenome‐based predictions should be interpreted with caution and integrative approaches combining culture‐dependent methods.

## Author Contributions

**Hanseob Shin:** writing – original draft, methodology, software, formal analysis, visualisation, investigation. **Min Ki Jeon:** investigation, software, methodology, writing – review and editing. **Hor‐Gil Hur:** writing – review and editing, funding acquisition, supervision, investigation, conceptualisation, project administration, resources.

## Funding

This work was supported by the National Research Foundation of Korea (RS‐2023‐NR076613).

## Conflicts of Interest

The authors declare no conflicts of interest.

## Supporting information


**Table S1:** Primer list for detection of dominant *bla*_OXA_ genes.

## Data Availability

Raw sequencing data for each sample used in this study were deposited at the NCBI Sequence Read Archive (SRA) database in the FASTQ format and are available under the BioProject accession number PRJNA506137.

## References

[emi470327-bib-0001] Bastian, M. , S. Heymann , and M. Jacomy . 2009. “Gephi: An Open Source Software for Exploring and Manipulating Networks.” Icwsm 8: 361–362.

[emi470327-bib-0002] Derya, A.‐A. , D. Aytan‐Aktug , P. T. L. C. Clausen , et al. 2022. “PlasmidHostFinder: Prediction of Plasmid Hosts Using Random Forest.” mSystems 7: e01180‐21. 10.1128/msystems.01180-21. 35382558 PMC9040769

[emi470327-bib-0003] Ellabaan, M. M. H. , C. Munck , A. Porse , L. Imamovic , and M. O. A. Sommer . 2021. “Forecasting the Dissemination of Antibiotic Resistance Genes Across Bacterial Genomes.” Nature Communications 12: 2435. 10.1038/s41467-021-22757-1. PMC8065159 33893312

[emi470327-bib-0004] Feng, T. , X. Chen , S. Wu , et al. 2025. “Predicting the Bacterial Host Range of Plasmid Genomes Using the Language Model‐Based One‐Class Support Vector Machine Algorithm.” Microbial Genomics 11: 1–9. 10.1099/mgen.0.001355. PMC12282233 39932495

[emi470327-bib-0005] Fitzpatrick, D. , and F. Walsh . 2016. “Antibiotic Resistance Genes Across a Wide Variety of Metagenomes.” FEMS Microbiology Ecology 92: fiv168. 10.1093/femsec/fiv168. 26738556

[emi470327-bib-0006] Hamidian, M. , J. J. Kenyon , K. E. Holt , D. Pickard , and R. M. Hall . 2014. “A Conjugative Plasmid Carrying the Carbapenem Resistance Gene blaOXA‐23 in AbaR4 in an Extensively Resistant GC1 *Acinetobacter baumannii* Isolate.” Journal of Antimicrobial Chemotherapy 69: 2625–2628. 10.1093/jac/dku188. 24907141 PMC4164139

[emi470327-bib-0007] Hyatt, D. , G. L. Chen , P. F. LoCascio , M. L. Land , F. W. Larimer , and L. J. Hauser . 2010. “Prodigal: Prokaryotic Gene Recognition and Translation Initiation Site Identification.” BMC Bioinformatics 11: 119. 10.1186/1471-2105-11-119. 20211023 PMC2848648

[emi470327-bib-0008] Ji, Y. , J. Shang , X. Tang , and Y. Sun . 2023. “HOTSPOT: Hierarchical Host Prediction for Assembled Plasmid Contigs With Transformer.” Bioinformatics 39: btad283. 10.1093/bioinformatics/btad283. 37086432 PMC10159655

[emi470327-bib-0009] Kanger, K. , N. G. H. Guilford , H. W. Lee , et al. 2019. “Antibiotic Resistome and Microbial Community Structure During Anaerobic Co‐Digestion of Food Waste, Paper and Cardboard.” bioRxiv: 564823. 10.1101/564823. 31922542

[emi470327-bib-0010] Li, D. , C. M. Liu , R. Luo , K. Sadakane , and T. W. Lam . 2015. “MEGAHIT: An Ultra‐Fast Single‐Node Solution for Large and Complex Metagenomics Assembly via Succinct de Bruijn Graph.” Bioinformatics 31: 1674–1676. 10.1093/bioinformatics/btv033. 25609793

[emi470327-bib-0011] Li, P.‐E. , C. C. Lo , J. J. Anderson , et al. 2017. “Enabling the Democratization of the Genomics Revolution With a Fully Integrated Web‐Based Bioinformatics Platform.” Nucleic Acids Research 45: 67–80. 10.1093/nar/gkw1027. 27899609 PMC5224473

[emi470327-bib-0012] Lo, C.‐C. , and P. S. G. Chain . 2014. “Rapid Evaluation and Quality Control of Next Generation Sequencing Data With FaQCs.” BMC Bioinformatics 15: 366. 10.1186/s12859-014-0366-2. 25408143 PMC4246454

[emi470327-bib-0013] Maurya, A. P. , D. Dhar , M. K. Basumatary , et al. 2017. “Expansion of Highly Stable blaOXA‐10 β‐Lactamase Family Within Diverse Host Range Among Nosocomial Isolates of Gram‐Negative Bacilli Within a Tertiary Referral Hospital of Northeast India.” BMC Research Notes 10: 145. 10.1186/s13104-017-2467-2. 28376860 PMC5379701

[emi470327-bib-0014] Menzel, P. , K. L. Ng , and A. Krogh . 2016. “Fast and Sensitive Taxonomic Classification for Metagenomics With Kaiju.” Nature Communications 7: 11257. 10.1038/ncomms11257. PMC4833860 27071849

[emi470327-bib-0015] Naas, T. , and P. Nordmann . 1999. “OXA‐Type β‐Lactamases.” Current Pharmaceutical Design 5: 865–879. 10539993

[emi470327-bib-0016] Pandey, D. , N. Singhal , and M. Kumar . 2021. “Investigating the OXA Variants of ESKAPE Pathogens.” Antibiotics 10: 1539. 34943751 10.3390/antibiotics10121539 PMC8699015

[emi470327-bib-0017] Raza, S. , H. Shin , H. G. Hur , and T. Unno . 2022. “Higher Abundance of Core Antimicrobial Resistant Genes in Effluent From Wastewater Treatment Plants.” Water Research 208: 117882. 10.1016/j.watres.2021.117882. 34837814

[emi470327-bib-0018] Risely, A. , A. Newbury , T. Stalder , et al. 2024. “Host‐Plasmid Network Structure in Wastewater Is Linked to Antimicrobial Resistance Genes.” Nature Communications 15: 555. 10.1038/s41467-024-44827-w. PMC10791616 38228585

[emi470327-bib-0019] Shin, H. , Y. Kim , D. Han , et al. 2021. “Emergence of High Level Carbapenem and Extensively Drug Resistant *Escherichia coli* ST746 Producing NDM‐5 in Influent of Wastewater Treatment Plant, Seoul, South Korea.” Frontiers in Microbiology 12: 1–7. 10.3389/fmicb.2021.645411. PMC8021692 33833746

[emi470327-bib-0020] Shin, H. , Y. Kim , S. Raza , T. Unno , S. H. Ryu , and H. G. Hur . 2022. “Dynamics of Genotypic and Phenotypic Antibiotic Resistance in a Conventional Wastewater Treatment Plant in 2 Years.” Frontiers in Microbiology 13: 898339. 10.3389/fmicb.2022.898339. 36033841 PMC9403409

[emi470327-bib-0021] Shin, H. , Y. Kim , S. Han , and H. G. Hur . 2023. “Resistome Study in Aquatic Environments.” Journal of Microbiology and Biotechnology 33: 277–287. 10.4014/jmb.2210.10044. 36655280 PMC10084755

[emi470327-bib-0022] Shin, H. , Y. Kim , and H.‐G. Hur . 2023. “Differentiation of Environment and Wastewater Treatment Plants by Core Antibiotic Resistance Genes and aadA2 as Indicators in South Korea.” Ecological Indicators 157: 111259. 10.1016/j.ecolind.2023.111259.

[emi470327-bib-0023] Su, Z. , A. Z. Gu , D. Wen , et al. 2025. “Rapid Identification of Antibiotic Resistance Gene Hosts by Prescreening ARG‐Like Reads.” Environmental Science and Ecotechnology 23: 100502. 10.1016/j.ese.2024.100502. 40059905 PMC11889379

[emi470327-bib-0024] Wayne, P. A. 2010. “Clinical and Laboratory Standards Institute: Performance Standards for Antimicrobial Susceptibility Testing: 20th Informational Supplement.” CLSI Doc M100‐S20.

